# Pan-Echinocandin Resistant *C. parapsilosis* Harboring an F652S Fks1 Alteration in a Patient with Prolonged Echinocandin Therapy

**DOI:** 10.3390/jof8090931

**Published:** 2022-09-01

**Authors:** Maria Siopi, Antonios Papadopoulos, Anastasia Spiliopoulou, Fotini Paliogianni, Nissrine Abou-Chakra, Maiken Cavling Arendrup, Christina Damoulari, Georgios Tsioulos, Efthymia Giannitsioti, Frantzeska Frantzeskaki, Iraklis Tsangaris, Spyros Pournaras, Joseph Meletiadis

**Affiliations:** 1 Clinical Microbiology Laboratory, Attikon University General Hospital, Medical School, National and Kapodistrian University of Athens, 12462 Athens, Greece; 2 4th Department of Internal Medicine, Attikon University General Hospital, Medical School, National and Kapodistrian University of Athens, 12462 Athens, Greece; 3 Department of Microbiology, University General Hospital of Patras, University of Patras Medical School, 26504 Patras, Greece; 4 Unit of Mycology, Statens Serum Institute, DK-2300 Copenhagen, Denmark; 5 Department of Clinical Microbiology, Rigshospitalet, DK-2100 Copenhagen, Denmark; 6 Department of Clinical Medicine, Faculty of Health Science, University of Copenhagen, DK-2100 Copenhagen, Denmark; 7 2nd Department of Critical Care, Attikon University General Hospital, National and Kapodistrian University of Athens, 12462 Athens, Greece

**Keywords:** *Candida parapsilosis*, echinocandin, acquired resistance, *FKS1* mutation

## Abstract

The isolation of a pan-echinocandin-resistant *Candida parapsilosis* strain (anidulafungin, caspofungin, micafungin and rezafungin EUCAST MICs > 8 mg/L) from urine of a patient following prolonged exposure to echinocandins (38 days of micafungin followed by 16 days of anidulafungin) is described. The isolate harbored the novel alteration F652S in the hotspot 1 region of fks1. Isogenic *C. parapsilosis* bloodstream isolates collected up to 1.5 months earlier from the same patient were susceptible to echinocandins (anidulafungin, caspofungin and micafungin EUCAST MICs 1–2, 1 and 1 mg/L, respectively) and contained wild-type *FKS1* sequences. This is the first report of pan-echinocandin resistance in *C. parapsilosis* associated with an aminoacid change in hotspot 1 region of fks1.

## 1. Introduction

*C. parapsilosis* is the second or third most prevalent cause of invasive candidiasis, depending on the geographic area and patient population [1]. In recent years, it has even surpassed *C. albicans* and become the predominant pathogen in some countries, including Greece [2], South Africa [3] and several Latin American countries [4,5,6]. *C. parapsilosis* is part of the colonizing skin flora and has propensity to form tenacious biofilms on medically implanted devices [1]. Horizontal transmission and nosocomial cluster outbreaks via contaminated medical equipment, inanimate environmental surfaces and hands of healthcare personnel are not uncommon [1].

Echinocandins are currently indicated as a first-line treatment option for invasive candidiasis [7,8]. *C. parapsilosis* is less susceptible in vitro to echinocandins compared to other common *Candida* spp. due to a naturally occurring polymorphism (P660A) in the highly conserved hotspot (HS) 1 region of the echinocandin target protein fks1 [9]. Persistence and breakthrough cases have been associated with echinocandin therapy for *C. parapsilosis* infections [10,11]. Nevertheless, most patients with systemic infections due to *C. parapsilosis* respond to echinocandin therapy [12], possibly due to its lower virulence [13]. Repeated exposure to echinocandin-class drugs is proposed to be a risk factor for developing resistance [14,15,16]. There are numerous publications on *FKS* mutant isolates with most alterations being associated with elevated MICs. However, *FKS* mutations associated with elevated echinocandin MICs have rarely been reported in *C. parapsilosis* [17].

Herein, we report the isolation of a pan-echinocandin-resistant *C. parapsilosis* urinary strain from a patient following prolonged echinocandin treatment for *C. parapsilosis* candidaemia, which harbored the fks1 aminoacid change F652S.

## 2. Case Report

### 2.1. Clinical Case

A 34-year-old 70 kg female (body mass index 24.22), was admitted to Attikon University Hospital (AUH) due to recommendation for electroconvulsive therapy. Prior to her initial admission, she had an extended (3.5 months) hospitalization at University Hospital of Patras (UHP) due to mental retardation, hypothyroidism, neuroleptic malignant syndrome, catatonia, refusal to eat or drink and prolonged fever of unknown origin. She had an indwelling urinary catheter (IUC), nasogastric tube and peripherally inserted central catheter (PICC) line in the right femoral vein.

**Figure 1:**
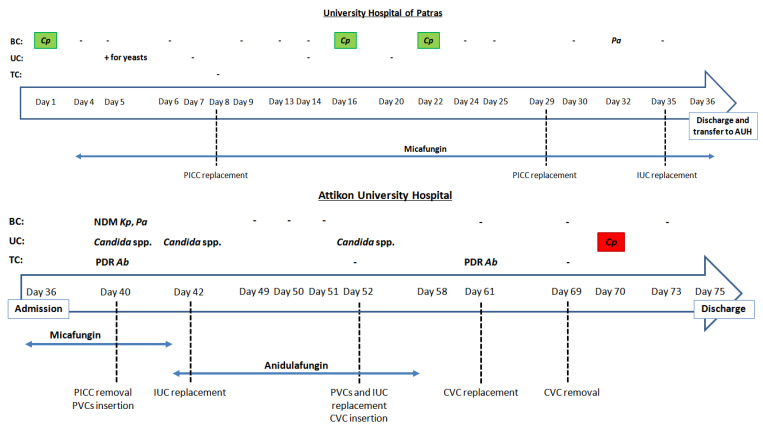
Timeline of microbiological investigation and antifungal therapy. Echinocandin-susceptible and echinocandin-resistant *C. parapsilosis* isolates are shaded in green and red, respectively. BC: blood culture; UC: urine culture; TC: tip culture; *Cp*: *C. parapsilosis*; *Pa*: *Pseudomonas aeruginosa*; *Kp*: *Klebsiella pneumoniae*; NDM: New Delhi metallo-β-lactamase; *Ab*: *Acinetobacter baumannii*; PDR: pan-drug-resistant; PICC: peripherally inserted central catheter; PVC: peripheral venous catheter; CVC: central venous catheter; IUC: indwelling urinary catheter.

During hospitalization at the UHP, the patient developed candidemia, with day 1 corresponding to the day of collection of the blood culture sample that was positive for yeast. The bloodstream isolate (N293) was identified as *C. parapsilosis* (VITEK 2 Compact automated system, BioMeriéux, Marcy l’Etoile, France) and antifungal susceptibility testing using gradient concentration strips (Etest, BioMeriéux, Marcy l’Etoile, France) revealed that it was susceptible to amphotericin B and echinocandins, but not to azoles (including fluconazole; FLC). Based on these findings, micafungin (MFG; 100 mg q24h) was administered according to the physicians’ decision on day 4. On day 5, her urine culture was positive for yeasts, identification of the isolate was not requested, and a repeat culture was recommended by the laboratory. On days 7, 14 and 20, the repeat urine cultures were negative. Repeated blood cultures until day 16 and culture of the catheter tip on day 7 were negative. On days 16 and 22, the fever persisted despite MFG therapy and blood cultures were positive for *C. parapsilosis* isolates N308 and N315 with the same Etest susceptibility profile as the N293. Repeated following blood cultures were negative for *Candida* (Figure 1).

On day 36, the patient was transferred to the AUH where she received parenteral nutrition. On day 42, MFG (day 38 of treatment) was replaced by anidulafungin (AFG; 100 mg q24h) due to sepsis-associated hypoxic hepatitis (SGOT 9898 IU/L, SGPT 2015 IU/L) taking into account the previously reported azole-resistant candidemia during her hospitalization at the UHP. No signs of endocarditis or septic thrombophlebitis were found. Urine cultures were positive for *Candida* spp. (>10^5^ cfu/mL) on day 40, 42 and 52 with urinalysis showing leukocyte esterase 75–500 U/mL, white blood cells 2–6/hpf and many yeast cells. During days 53–57, the patient was afebrile, respiratory and hemodynamic stable and thus the physicians decided to discontinue AFG (day 16 of treatment). On day 70, urinalysis showed leukocyte esterase 250 U/mL, white blood cells 40–50/hpf and many yeast cells, whereas the urine culture yielded *C. parapsilosis* (>10^5^ cfu/mL) resistant to all ehinocandins, but not azoles (details are described below). On day 75, her close relatives decided to transfer her to a private-sector hospital. During her hospitalization, PICCs, central/peripheral venous catheters (CVCs/PVCs) and IUCs were replaced several times, blood cultures were positive for multidrug-resistant gram-negative bacteria (Figure 1) and different antibiotic agents were administered.

### 2.2. Mycological Workup

Frozen stocks of the three bloodstream strains were retrospectively forwarded from the UHP to the microbiology laboratory of AUH for detailed mycological workup. Isolates N293, N308, N315 (bloodstream) and AUH1957 (urinary) were identified to the species level using MALDI-TOF mass spectrometry (Bruker Daltonics, Bremen, Germany). Species identification was verified by sequencing the ITS1-5.8S-ITS2 region using ITS1 (5-TCCGTAGGTGAACCTGCGG-3) and ITS4 (5-TCCTCCGCTTATTGATATGC-3) primers [18]. Antifungal susceptibility testing was performed following the EUCAST broth microdilution (BMD) reference methodology E. Def 7.3.2 [19]. All isolates were susceptible to amphotericin B (MIC 0.25–0.5 mg/L). Whereas the bloodstream isolates were echinocandin-susceptible (AFG, caspofungin (CAS) and MFG MICs 1–2, 1 and 1 mg/L, respectively), the urinary strain exhibited a pan-echinocandin-resistant phenotype (AFG, CAS, MFG and rezafungin MICs > 8 mg/L). Of note, azole resistance previously reported for the bloodstream isolates was not confirmed by the EUCAST reference BMD method. In particular, all three isolates were found susceptible to FLC (MIC 2 mg/L), voriconazole (MIC 0.03 mg/L), itraconazole (MIC 0.03–0.06 mg/L) and posaconazole (MIC 0.016–0.03 mg/L). Similarly, the urinary strain was azole-susceptible (FLC, voriconazole, itraconazole and posaconazole MIC 2, 0.03, 0.06 and 0.03 mg/L, respectively) (Table 1). Of note, resistance to echinocandins had not previously been detected in other clinical *C. parapsilosis* strains in our hospital [20].

*FKS1*-HS sequencing of the *C. parapsilosis* isolates was performed as previously described [21]. All bloodstream strains displayed wild-type *FKS1* (HSs) sequences. As regards the echinocandin-resistant urinary isolate, a mutation was detected in the HS1 region of the *FKS1* leading to replacement of phenylalanine by serine at position 652 (F652S), whereas it was wild-type for HS2 (Table 1).

In order to assess the genotypic diversity among the *C. parapsilosis* isolates microsatellite typing with six short tandem repeat markers was performed as previously described [22]. In addition, four isolates from four random Danish patients were included as comparators. Microsatellite typing revealed that the four isolates from the case patient as well as an isolate from a nonrelated Danish patient shared the same genotype (13-13-17-17-25-25-10-10-10-10-10-10), whereas the remaining three Danish isolates were unique and different from the case patient isolates (genotypes 7-14-28-28-25-30-14-14-10-10-10-10, 10-22-17-17-24-24-10-10-11-11-10-10 and 14-14-17-17-26-26-10-10-10-10-10-10, respectively) (Table 1).

## 3. Discussions

As echinocandin usage broadens, the emergence of resistance represents a critical threat to patient management and clinical success. To the best of our knowledge, this is the first report on in vivo-acquired pan-echinocandin resistance associated with a target gene HS mutation in *C. parapsilosis*. The isolate was recovered from a urine sample of a patient following 54 days of echinocandin administration (38 days of MFG followed by 16 days of AFG). It harbored an aminoacid alteration (F652S) in the HS1 region of fks1 protein not previously reported in *C. parapsilosis* and not found in the isogenic echinocandin-susceptible and *FKS1* wild-type bloodstream isolates collected up to 1.5 months earlier from the same patient.

Risk factors for candidemia/candiduria are prolonged hospitalization, foreign bodies (PICC, CVC, IUC), use of broad-spectrum antibiotics, and parenteral nutrition, all of which were present in our patient [23,24]. Indeed, the major risk factor for developing *Candida* spp. breakthrough infections caused by isolates with echinocandin resistance-conferring *FKS* mutations is the exposure to echinocandins within the preceding ~100 (range 7–450) days [25]. Breakthrough echinocandin non-susceptible *C. parapsilosis* isolates (CLSI MIC > 2 mg/L) have been reported in severely immunosuppressed patients after a median (range) duration of contiguous exposure prior to infection of 19 (5–50) days, although no *FKS* mutations neither a pan-echinocandin-resistant phenotype were found [10,11,16]. In vitro, 19 (63%), 18 (60%) and 14 (47%) of *C. parapsilosis* isolates exposed to increasing concentrations of echinocandins up to 8 mg/L using serial passages were resistant to CAS, MFG and AFG, respectively, including 4 (13%) that were cross-resistance to all three echinocandins by the end of the experiment (60 exposures, 270 days) [15]. The first resistant strains were observed after 21 exposures (49 days) of CAS and MFG and after 25 exposures (63 days) of AFG, durations that are similar to the exposure in our patient before the resistant strain was cultured.

Fungal cells located at sites of colonization or deep-seated tissue infections exposed to lower drug concentrations may provide a potential nest and reservoir of *FKS*-mediated resistance, as previously described in a murine model of gastrointestinal colonization [26] and in the oral flora of patients post candidemia treatment [27]. Echinocandin therapy eliminated the isogenic susceptible *C. parapsilosis* isolate from the blood but failed to sterilize urine. We hypothesized that the lack of renal excretion and associated low levels of echinocandins in urine [24] in combination with the high microbial burden during candiduria and the presence of IUC facilitating biofilm formation provided a niche for emergence of resistance [28]. This pathogenesishas previously been reported in cases of echinocandin resistance mediated by mutations in the *FKS2* gene in *C. glabrata* urinary isolates [29].

Echinocandin resistance is usually due to mutations in two highly conserved HS regions of *FKS1* gene for all *Candida* spp. as well as *FKS2* gene for *C. glabrata*, which encode the drugs’ target (1–3)-β-D-glucan synthase [28,30]. To date, several point mutations have been characterized and associated with resistance in *Candida* spp. other than *C. parapsilosis* [31,32]. Most of these confer cross-resistance to all three echinocandins [30], similar to our case. The magnitude of the MIC elevation depends on the position and the nature of amino acid substitution, with the most pronounced echinocandin resistance phenotypes being attributable to alterations involving the first and fifth amino acid (phenylalanine (F) and serine (S)) in the HS1 region of *Candida* spp. [28,30]. Indeed, the F652S involves the first aminoacid of the HS1 region of the fks1 subunit in *C. parapsilosis* and is equivalent to F641S and F625S in *C. albicans* and *C. glabrata*, respectively. The first amino acid change in the HS1 region of fks2 in *C. glabrata* F659S has also been associated with elevated MIC of ibrexafungerp, a novel triterpenoid glucan synthase inhibitor [33].

Recently, Arastehfar et al. reported four MFG-resistant (CLSI MIC > 8 mg/L) *C. parapsilosis* bloodstream isolates carrying the mutation R658G in HS1 of fks1. This seventh codon in HS1 has been associated with weak mutation resulting in discrete MIC elevation. The first recovered isolate was susceptible to AFG and intermediate to CAS (CLSI MIC 2 and 4 mg/L, respectively), as opposed to ours, whereas it was obtained from a patient previously exposed to CAS, but detailed data on dosage and treatment duration were not available. The other three strains were collected in a time span of 11 months from echinocandin-naïve patients hospitalized in the same ward, were susceptible to both AFG and CAS (CLSI MICs 2 mg/L) and their close genetic relatedness implicated clonal expansion from a single lineage [17]. Interestingly, an upcoming replication study performed by Liang et al. failed to identify the presence of HS point mutations, including the R658G, in echinocandin-non-susceptible *C. parapsilosis* clinical isolates (CLSI MICs 4- ≥8 mg/L) [34], in line with previous reports [10,21,35]. This finding suggests that amino acid changes outside the well-known HS regions may likely contribute to the complexity of echinocandin resistance in *C. parapsilosis* [36], as was also recently described for *C. glabrata* [37], highlighting the importance of sequencing the entire *FKS* gene in cases with wild-type HS regions to characterize the resistant phenotype.

## 4. Conclusions

Taken together, the present case illustrates the importance of closely monitoring patients with *C. parapsilosis* infections receiving long-term echinocandin therapy as development of resistance may occur. This is particularly important since the current armamentarium of antifungals in clinical use is limited and since FLC-resistant *C. parapsilosis* is emerging in multiple countries worldwide [2,3,4,38]. Due to the propensity of *C. parapsilosis* to cause outbreaks, an outbreak by an azole- and echinocandin-resistant isolate could resemble the epidemic of *C. auris* with limited therapeutic options. Although similar amino acid substitutions in fks1 HS regions in other *Candida* spp. suggest that this is the cause of echinocandin resistance, confirmation with directed mutation experiments is required for the newly identified F652S in *C. parapsilosis*. Further studies to better understand the fitness impact of this mutation and how *C. parapsilosis* is adapting to echinocandin exposure in vivo are warranted.

## Figures and Tables

**Table 1:** Clinical and microbiological data of *C. parapsilosis* isolates.

Isolate	Clinical Specimen	Antifungal Treatment, Duration in Days	EUCAST MIC (mg/L)										Fks1Alteration		Genotype
			AMB	FLC	VRC	ITC	POS	ISA	AFG	CAS	MFG	RZF	HS1	HS2	
N293	Blood	None	0.5	2	0.03	0.03	0.016	0.016	2	1	1	ND	None	None	Same **^a^**
N308	Blood	MFG, 13 days	0.5	2	0.03	0.06	0.03	0.016	1	1	1	ND	None	None	Same **^a^**
N315	Blood	MFG, 19 days	0.25	2	0.03	0.06	0.016	0.016	1	1	1	ND	None	None	Same **^a^**
AUH1957	Urine	MFG, 38 days + AFG, 16 days	0.25	2	0.03	0.06	0.03	0.016	>8	>8	>8	>8	F652S	None	Same **^a^**

**^a^** The genotype for 3AI-3AII-3BI-3BII-3CI-3CII-6AI-6AII-6BI-6BII-6CI-6CII loci was 13-13-17-17-25-25-10-10-10-10-10-10; AMB: amphotericin B; FLC: fluconazole; VRC: voriconazole; ITC: itraconazole; POS: posaconazole; ISA: isavuconazole; AFG: anidulafungin; CAS: caspofungin; MFG: micafungin; RZF: rezafungin; ND: not determined; HS: hot spot.

## Data Availability

All data presented in this study are included in the article.
